# The effect of spaced learning on the learning outcome and retention of nurse anesthesia students: a randomized-controlled study

**DOI:** 10.1186/s12909-024-05290-9

**Published:** 2024-03-21

**Authors:** Ali Khalafi, Zahra Fallah, Hamid Sharif-Nia

**Affiliations:** 1 Department of Anesthesiology, School of Allied Medical Sciences, Ahvaz Jundishapur University of Medical Sciences, Ahvaz, Iran; 2 Department of Anesthesiology, School of Allied Medical Sciences, Ahvaz Jundishapur University of Medical Sciences, Ahvaz, Iran; 3 Education Development Center, Mazandaran University of Medical Sciences, Sari, Iran; 4 Department of Nursing, Amol School of Nursing and Midwifery, Mazandaran University of Medical Sciences, Sari, Iran

**Keywords:** Spaced learning, Long-term memory, Education, Anesthesia

## Abstract

**Background:**

Poor learning and retention are common problems of students, which may be alleviated by optimization of widely used educational methods such as lectures. This study aimed to investigate the effect of spaced learning on the learning outcome and retention of nurse anesthesia students.

**Methods:**

This was a randomized controlled study with a pre-and post-test design on 64 nurse anesthesia students who were divided into two groups of spaced lecture (*n* = 32) and conventional lecture (*n* = 32). The spaced lectures included three 30-minute training sessions with 10-minute intervals while the conventional sessions including 90 min of continuous training. Students’ knowledge was measured using one valid and reliable questionnaire developed by the research team. All students in both groups took a pre-test, and their level of knowledge acquisition was evaluated immediately after the training. Their level of knowledge retention was tested two and four weeks after the lecture.

**Results:**

There was no significant difference between the two groups regarding demographic characteristics (*p* > 0.05). In the pre-test, the mean score of knowledge in the intervention group was lower than that in the control group, there was no significant difference (*p* = 0.177). But after the intervention, the mean scores of learning outcome and retention in the intervention group were significantly higher than those in the control group (*p* < 0.001, eta = 0.576). Also, the results showed that learning outcome and retention across the three academic semesters in the two groups are significantly different, and students with a higher academic semester obtained a significantly higher mean score of knowledge and retention (*p* < 0.001, eta = 0.604).

**Conclusion:**

Spaced learning improves nurse anesthesia students’ knowledge and retention more than conventional method. Future studies focusing on spaced learning should specifically examine the impact of duration and number of intervals, as well as the time gap between training and measurement of learning retention.

## Introduction

Memory formation is greatly influenced by temporal features of stimulus presentation. Spaced learning, which involves temporally distributed learning with resting intervals, is known to be more efficient than “massed” learning which involves no resting intervals [1]. The spaced learning sessions can play an essential role in memory retention. This has come to be known in the literature as the “spacing effect”, which can exist in different time intervals ranging from minutes to months [2]. The spacing effect is simply based on the principle that when learning breaks into several sessions of shorter durations, with spaced intervals in between, acquisition of knowledge will be promoted, and leaners will be less likely to forget that piece of information [3]. More than a century ago, Ebbinghaus introduced the spacing effect in the field of psychology [1], and since then, many studies have focused on the effectiveness of spaced learning [4–6]. It has been proven that better outcomes will be achieved if the educational content is presented in a learning process which involves repetition for a second or third time after one or more diverse intervals from the first encounter (spaced learning), as opposed to a state in which the second set of information immediately follows the first in a bolus or mass presentation [7].

The principle of spaced learning is supported by evidence from neuroscience and cognitive psychology [8]. But, Exploring the specific details of the “spacing effect” applications is rather challenging due to the absence of vital information on used spacing formats such as the number and duration of intervals between educational encounters, the duration of the retention interval, and the number and duration of learning sessions. Notably, less research is conducted on the benefits of spaced learning in the instructional phase that is during teaching [9]. Psychological and neuroscientific research findings on the mechanisms of memory formation suggest that spaced learning also works using shorter intervals. Therefore, applying spaced learning on the timescale of minutes to hours may have implications for current massed learning in classroom settings, such as conventional lectures, which still holds a prominent position in health professions education worldwide [2]. The advantages of spaced learning include less mental fatigue, more acquisition and learning, higher attractiveness, longer retention, wasting no time, reduced stress for learners, and a pleasant learning experience [10]. The beneficial effects of spaced learning on memory have also been shown in a variety of learning tasks related to real [11], conceptual [12], and procedural knowledge [13].

Given the wide scope of medical sciences and the ever-increasing advancements in medical knowledge, universities of medical sciences must find efficient ways to help students master the knowledge they are supposed to acquire [14]. Meanwhile, in the operating room (OR), as a unit where speed and quality of services are of paramount importance, special attention should be paid to knowledge retention [3]. Anesthesia teams consisting of anesthesiologists and nurse anesthetists provide a wide range of perioperative services which often expose them to hazardous events [15]. They need rapid decision-making in response to the physiological reactions of patients and unexpected surgical events; for example, diagnosing problems like malignant hyperthermia requires more coordination of information [16]. Moreover, they use techniques that require advanced knowledge, critical thinking, and clinical expertise [17, 18]. This highlights one of the goals of anesthesia education that is helping learners acquire, retain, recall, and apply knowledge [19, 20]. Therefore, to promote long-term knowledge retention of nurse anesthesia students, this study investigated the spaced presentation of 90-minute lectures as opposed to the conventional presentation. In other words, the present study aimed to analyze the impact of spaced learning on the learning outcomes and retention of nurse anesthesia students.

## Method

### Study design and setting

This randomized-controlled study with a pre-and-post-test design was conducted at Ahvaz Jundishapur University of Medical Sciences (AJUMS), Ahvaz, Iran, from October to November 2022.

### Participants

This study involved 64 nurse anesthesia students of AJUMS who were selected by the convenience sampling method from among the nursing anesthesia students who met the inclusion criteria. The inclusion criteria were: (1) nurse anesthesia students in 3rd, 5th or 7th semesters and (2) willingness to participate in research. The exclusion criteria were: (1) withdrawal from the study at any time or for any reason, (2) absence in a lecture session, and (3) inadequate questionnaire completion. In this study, the students were allocated to the intervention and control groups according to the academic year, using stratified randomization. Each student was randomly assigned a code, and the codes were then placed in three boxes according to the academic year. The first code extracted from each box was assigned to the intervention group, while the next code indicated allocation to the control group. This process continued until all students were selected.

### Data collection

Data were collected using a form consisting of two sections. The first section was devoted to demographic characteristics including age, gender, and the semester in which the students were studying.

The second section, the knowledge questionnaire, included 20 four-choice questions on anesthesia in neurosurgery. The questions were aimed to measure the students’ basic knowledge and their learning outcome and retention. Correct answers were scored 1, while incorrect ones were noted as 0. No negative marking was done for incorrect answers.

To confirm the validity of the knowledge questionnaire, the Messick validity framework was used [21, 22]. We assessed both content and face validity. In terms of content validity, we extracted questionnaire from articles [23, 24], and presented it to 10 faculty members from the Anesthesiology Department. Their task was to assess the relevance of each item. We then calculated the content validity rate (CVR) and content validity index (CVI) for each item, using the Lawshe table and adjusted Kappa coefficient as guidelines. To ensure face validity, we distributed the questionnaire to 10 students who met the study’s inclusion criteria but were not part of the study. We sought their opinions to make the necessary modifications to the questionnaire. Regarding the reliability of the knowledge instrument, we employed a test-retest method with a 10-day interval. Initially, 30 students who met the study’s inclusion criteria but were not part of the study completed the instrument, and after ten days, we handed the same instrument to the same students, and asked them to respond again. We then assessed the stability of the instrument using the intraclass correlation coefficient (ICC: 0.889).

### Intervention

Both groups attended a training session on the topic of anesthesia in neurosurgery. Anesthesia in neurosurgery requires an understanding of brain anatomy, physiological flow dynamics of the brain, possible changes that occur in response to pathologically increased intracranial pressure (ICP), and safe administration of anesthesia in neurosurgery [25]. It is a subject where the knowledge of the nurse anesthetist may directly influence patient outcome [26]. In AJUMS, this subject is taught at the end of the 5th semester. Therefore, this topic was new for 3rd and 5th semester students, and a year ago this topic was taught to 7th semester students. In order to increase the sample size, we also used 7th semester students. Since this topic is relatively difficult and has a lot of cognitive load [24, 26], the students of the 7th semester had forgotten many of the theoretical content and repeating the content could be helpful.

The content of this session which was extracted from reference books of anesthesia and the latest relevant articles [27–29] was approved by 10 professors of anesthesia and anesthesiologists in terms of validity, significance, and relevance. The lectures included a brief introduction to brain anatomy, neurophysiology, ICP, anesthesia in supratentorial brain space-occupying lesions, anesthesia in infratentorial or posterior fossa space-occupying lesions, and anesthesia in brain aneurysm surgery and spinal cord injury. Spaced and conventional lectures were held by a lecturer who was a faculty member of the Anesthesiology Department at AJUMS with more than 10 years of teaching experience in the topic of anesthesia in neurosurgery. In both lectures, the same PowerPoint slides were used. Thus, both intervention and control groups received training using the same content.

Spaced lecture: The total presentation time of 90 min was divided into three teaching sections of approximately 30 min each, separated by breaks (10-minutes inter-study intervals), resulting in a 110-minutes lecture. To incorporate repetition as the second key element in spaced learning, each break began with a brief review of the information presented in the previous section to activate the students’ pre-organizers so that they could link the new information to be acquired to the information they already acquired in the previous section. To this aim, the instructor used 2–3 summary slides that covered the most essential points. The 10-minute intervals were based on our interpretation of neuroscience studies. During the 10-minutes breaks, the students were engaged in distractor activities, such as talking to their classmates and going outside the classroom [30].

Conventional lecture: In the control group, the instructor taught the whole 90 min non-stop.

The method and sequence of measuring knowledge in the intervention and control groups were as follows: All students in both groups took a pre-test and the level of knowledge acquisition was evaluated immediately after the training. Their level of knowledge retention was tested two and four weeks after the lecture.

### Statistical analysis

After data collection, data were imported into IBM SPSS Statistics for Windows, Version 21.0. Armonk, NY: IBM Corp. Descriptive statistics such as mean (standard deviation) and number (percent) were used to present the quantitative and qualitative variables, respectively. The normal distribution of the data in the two groups was evaluated using the Shapiro-Wilks test, and based on the normal distribution of data, the repeated measures analysis of variance (ANOVA) was used to analyze the research hypotheses. The significance level was considered less than 0.05.

## Results

The results showed that most of the participants were women (*N* = 45, 70.3%) and studying in the 3rd semester (*N* = 29, 45.3%) along with 24 (37.5%) studying in the fifth and 11 (17.2%) in the seventh semester. The mean age of the participants was 21.67 ± 2.65 years. Most of the participants in the intervention and control groups were females (25 (78.1%) versus 20 (62.5%), respectively), and there was no significant difference between the two groups regarding gender. To compare the effect of spaced learning method in the intervention group with that of the conventional education in the control group, repeated measures ANOVA was used. First, Mauchly’s sphericity test was used to evaluate the scores obtained from the two groups in four rounds (i.e., pre-test, immediate post-test, two-week post-test, and four-week post-test). Due to the significance of Mauchly’s sphericity test (*p* < 0.001), Greenhouse-Geisser results were taken into account.

Table 1 shows the mean knowledge scores of anesthesia in neurosurgery in the different groups of nurse anesthesia students at different measurement times. Although the mean scores in the pre-test showed that the mean score of basic knowledge in the intervention group was lower than that in the control group (spaced learning: 7.28 ± 2.331 vs. conventional: 8.16 ± 2.77), there was no significant difference (*p* = 0.177). The mean scores of learning outcomes and retention in the intervention group were significantly higher than those in the control group (*p* < 0.001, eta = 0.576). Also, the results were significantly different in the two groups regardless of the time trend (*p* < 0.001, eta = 0.514). The effect sizes of 0.576 and 0.514 suggest a moderate positive linear relationship between the variables under investigation. Finally, examining the effect of time trends on learning outcomes and retention revealed that there is a significant difference between the different testing rounds the students were tested in (*p* < 0.001, eta = 0.870). The effect size of eta = 0.870 suggests a robust positive linear relationship between the variables under investigation. This indicates a strong correlation between the variables, demonstrating a clear and significant connection between them. The results showed that learning outcomes and retention across the three academic semesters in the two educational methods are significantly different (*p* < 0.001, eta = 0.604). The effect size of eta = 0.640 indicates a significant portion of the variance. This suggests that a large proportion of the variability in the data can be attributed to the factor being studied. The results were also significantly different from each other regardless of the time trend in groups (*p* < 0.001, eta = 0.238). An effect size of eta = 0.238 indicates that a moderate amount of the variance for the dependent variable. Finally, examining the effect of the time trend on learning outcomes and retention showed that there is a significant difference between different measurement rounds (*p* < 0.001, eta = 0.873). An effect size of eta = 0.8738 suggests that a large proportion of the variance in the dependent variable can be explained by the independent variable studied.

**Table 1:** The mean and standard deviation of the learning outcome and retention score in the different groups of nurse anesthesia students at different measurement times

		Measurement round						
		Pre-test	First round (immediate)	Second round (after two weeks)	Third round (after four weeks)	*p*-value (effect size)*		
**Semester**	**Group**	**Mean ± SD**				**Time**	**Group**	**Time-Group**
3rd semester	Intervention	6.80 ± 1.207	17.73 ± 0.884	16.07 ± 1.033	14.33 ± 0.976	< 0.001 (0.873)	< 0.001 (0.238)	< 0.001 (0.604)
	Control	6.71 ± 1.490	13.07 ± 2.40	8.71 ± 1.437	7.86 ± 1.406			
5th semester	Intervention	6.83 ± 2.082	18.75 ± 0.965	17.75 ± 1.138	17.25 ± 1.35			
	Control	7.75 ± 1.288	15.00 ± 1.80	12.42 ± 2.314	10.33 ± 2.22			
7th semester	Intervention	9.80 ± 3.962	18.80 ± 1.789	17.60 ± 1.673	16.60 ± 1.51			
	Control	12.33 ± 3.32	15.50 ± 2.07	13.67 ± 2.251	12.33 ± 3.01			
Total	Intervention	7.28 ± 2.331	18.28 ± 1.17	16.94 ± 1.413	15.78 ± 1.82	< 0.001 (0.870)	< 0.001 (0.514)	< 0.001 (0.576)
	Control	8.16 ± 2.77	14.25 ± 2.32	11.03 ± 2.845	9.62 ± 2.661			

*Repeated measure ANOVA

## Discussion

To the best of our knowledge, this is the first study to investigate the effect of spaced learning on learning outcomes and retention of nurse anesthesia students. To test our hypothesis, we used a randomized-controlled design in which we compared a spaced lecture with a conventional one. The findings showed that the mean scores of acquisition and retention of knowledge in the intervention group were significantly higher than those in the control group. Also, the mean scores of the 7th-semester students in both groups were higher compared with the 3rd and 5th-semester students, which can be attributed to the fact that the 7th-semester students had already received education on anesthesia in neurosurgery one year before the commencement of our study.

The findings of the present research are consistent with the results of previous studies [31–34]. In a study in India, Chugh and Tripathi (2020) investigated the effect of spaced education of a health-related topic on improving knowledge retention in medical students. Their results indicated that repetitions and spaced tests caused significant improvements in student learning and retention [32]. Dabiri et al. conducted a study in Iran that investigated the effect of test-enhanced spaced learning on the examinations of the otolaryngology board as well as annual residency examinations. They concluded that test-enhanced spaced learning may be useful in the clinical education environment to improve learning outcomes [35]. Also, Timmer et al. (2020) investigated the effect of spaced instruction on knowledge retention in medical education in the Netherlands. In their study, they included short 5-minute intervals between lecture sessions to enhance the process of memory formation. However, no beneficial effects on knowledge retention were found, suggesting that the 5-min intervals may be too short to stimulate the consolidation process [33].

In the current study, short 10-minute intervals were included between lecture sessions and seemed to be effective because Kelley and Whatson also obtained positive results in their spaced learning strategy in classroom sessions with same short 10-minutes intervals. They also included physical distraction activities in their design because neuroscientists believe that this prevents cognitive interference in the memory formation process [36]. The present study used similar patterns in class management as used in Kelley and Whatson. However, care should be exercised in interpreting the results, as findings may be highly sensitive to the study design. For example, in this study, knowledge retention was measured 2 and 4 weeks after training, while Timmer et al. and Kelley and Whatson measured it 8 and 5 days after the intervention, respectively. Our time interval was chosen because the Ebbinghaus Curve of Forgetting shows that forgetting decreases exponentially, and most of the forgetting occurs in the first week after initial learning [33, 37]. Another notable difference between our study and the studies mentioned above is related to the fact that in this study, short summaries of the content of each section were included at the beginning of the next section to incorporate the key element of repetition in spaced training, whereas Kelley and Whatson repeated their 15-minute training three times. Our reason for choosing this design was that it was closer to the conventional teaching style and was easy to implement. Despite some empirical evidence, including the present study, researchers acknowledge that optimal spacing protocols for humans remain unknown, and attempts to optimize the spacing effect have usually been based on trial and error [2]. Our study contributes to the literature by informing that 10-minute intervals in a lecture seem sufficient to promote knowledge acquisition and retention.

Despite its strengths, the present study also has limitations. Firstly, the small sample size and the fact that the study was conducted in a single center restrict the generalizability of the findings. To overcome this limitation, it is recommended to conduct further multicenter studies with a larger sample size. Additionally, it is important to note that the results of this study may not be applicable to all educational contexts. The generalization of these findings depends heavily on the clear specification of the characteristics of the specific context to which they are intended to be applied. Another limitation of this study is the potential influence of the pre-test on the post-test results. The students took a pre-test immediately before the training, which may have affected their performance on the post-test. To minimize this effect, it is suggested to introduce a longer time interval between the pre- and post-tests to accurately measure the learning outcome. A final limitation of this study was that due to the limited number of neurosurgery operations in AJUMS teaching hospitals, we could not measure the transfer of knowledge that learned in the classroom to the actual clinical setting. Since neuroanesthesia is a subject where the knowledge of the nurse anesthetist may directly influence patient outcome [2], comparing the knowledge application in the real work environment between two groups of spaced and conventional training can be beneficial.

The concept of spaced learning emphasizes that knowledge retention is enhanced when learning sessions are spaced out over time. By re-exposing learners to information at intervals, retention is more effective. This method has been proven to be highly effective in improving memory and understanding of the material. This approach has been recognized as beneficial in health professions education, including nurse anesthesia programs, where students often struggle to remember what they have learned [2]. In the realm of nurse anesthesia education, implementing spaced learning techniques like retrieval practice, interleaving, and distributed practice can greatly enhance long-term knowledge retention and improve learning outcomes [38]. Furthermore, the testing effect, which refers to the phenomenon where memory tests enhance long-term retention, provides additional evidence for the significance of evaluating the application of knowledge in nurse anesthesia education [39].

## Conclusion

The present study demonstrates that spaced learning improves nurse anesthesia students’ knowledge and retention more than conventional method. This study recommended that university instructors incorporate spaced learning methods into their classrooms to enhance learning outcomes and retention. Future studies focusing on spaced learning should specifically examine the impact of duration and number of intervals, as well as the time gap between training and measurement of learning retention. Furthermore, researchers can explore the combination of spaced learning with other effective strategies to further enhance knowledge acquisition and retention.

## Data Availability

The datasets used and/or analyzed during the current study are available from the corresponding author on reasonable request.
